# Mechanism of sensitization of MDR cancer cells by Pluronic block copolymers: Selective energy depletion

**DOI:** 10.1054/bjoc.2001.2165

**Published:** 2001-12

**Authors:** E V Batrakova, S Li, W F Elmquist, D W Miller, V Y Alakhov, A V Kabanov

**Affiliations:** College of Pharmacy, Department of Pharmaceutical Sciences, Nebraska Medical Center, 986025, Omaha, NE, 68198-6025, USA; Supratek Pharma Inc., 513 blvd. des Prairies, Case Postale 100, Laval, PQ, H7N 4Z3, Canada

**Keywords:** ATP, doxorubicin, MDR, Pluronic, sensitisation

## Abstract

This paper, for the first time, demonstrates that exposure of cells to the poly(ethylene oxide)-poly(propylene oxide) block copolymer, Pluronic P85, results in a substantial decrease in ATP levels selectively in MDR cells. Cells expressing high levels of functional P-glycoprotein (MCF-7/ADR, KBv; LLC-MDR1; Caco-2, bovine brain microvessel endothelial cells [BBMECs]) are highly responsive to Pluronic treatment, while cells with low levels of P-glycoprotein expression (MCF-7, KB, LLC-PK1, human umbilical vein endothelial cells [HUVECs] C2C12 myoblasts) are much less responsive to such treatment. Cytotoxicity studies suggest that Pluronic acts as a chemosensitizer and potentiates cytotoxic effects of doxorubicin in MDR cells. The ability of Pluronic to inhibit P-glycoprotein and sensitize MDR cells appears to be a result of ATP depletion. Because many mechanisms of drug resistance are energy dependent, a successful strategy for treating MDR cancer could be based on selective energy depletion in MDR cells. Therefore, the finding of the energy-depleting effects of Pluronic P85, in combination with its sensitization effects is of considerable theoretical and practical significance. © 2001 Cancer Research Campaign http://www.bjcancer.com


*British Journal of Cancer* (2001) **85**(12), 1987–1997
© 2001 Cancer Research Campaign
doi: 10.1054/ bjoc.2001.2165, available online at http://www.idealibrary.com on IDEAL

http://www.bjcancer.com

### Mechanism of sensitization of MDR cancer cells by Pluronic block copolymers: Selective energy depletion

**EV Batrakova[1], S Li[1], WF Elmquist[1], DW Miller[1], VY Alakhov[2] and AV Kabanov[1]**

[1]College of Pharmacy, Department of Pharmaceutical Sciences, 986025 Nebraska Medical Center, Omaha, NE 68198-6025, USA; [2]Supratek Pharma Inc., 513 blvd. des Prairies, Case Postale 100, Laval, PQ, Canada H7N 4Z3

**Summary** This paper, for the first time, demonstrates that exposure of cells to the poly(ethylene oxide)-poly(propylene oxide) block copolymer, Pluronic P85, results in a substantial decrease in ATP levels selectively in MDR cells. Cells expressing high levels of functional P-glycoprotein (MCF-7/ADR, KBv; LLC-MDR1; Caco-2, bovine brain microvessel endothelial cells [BBMECs]) are highly responsive to Pluronic treatment, while cells with low levels of P-glycoprotein expression (MCF-7, KB, LLC-PK1, human umbilical vein endothelial cells [HUVECs] C2C12 myoblasts) are much less responsive to such treatment. Cytotoxicity studies suggest that Pluronic acts as a chemosensitizer and potentiates cytotoxic effects of doxorubicin in MDR cells. The ability of Pluronic to inhibit P-glycoprotein and sensitize MDR cells appears to be a result of ATP depletion. Because many mechanisms of drug resistance are energy dependent, a successful strategy for treating MDR cancer could be based on selective energy depletion in MDR cells. Therefore, the finding of the energy-depleting effects of Pluronic P85, in combination with its sensitization effects is of considerable theoretical and practical significance. © 2001 Cancer Research Campaign http://www.bjcancer.com

**Keywords**: ATP; doxorubicin; MDR; Pluronic; sensitisation

Poly(ethylene oxide)-poly(propylene oxide) block co-polymers (Pluronic or 'poloxamer') have recently been used in formulations to treat drug-resistant cancers (Alakhov et al, 1996; Batrakova et al, 1996; Venne et al, 1996). Following validation using in vitro and in vivo models, a formulation that contains doxorubicin and Pluronic mixture (L61 and F127), SP1049C, is currently undergoing Phase I clinical trials (Alakhov et al, 1999). Experimental studies have demonstrated that Pluronic block copolymers sensitize MDR cells, resulting in an increase in the cytotoxic activity of anthracyclines and other cytotoxic drugs by 2–3 orders of magnitude (Alakhov et al, 1996; Venne et al, 1996). This finding is important in light of the major problem of drug resistance to antineoplastic agents, which severely limits the chemotherapy of many cancer tumours (Goldstein et al, 1992). Tumours with the MDR phenotype have been widely recognized as one of the most difficult types to treat. MDR cells overexpress efflux transporters belonging to a superfamily of ATP-binding cassette (ABC) proteins, such as P-glycoprotein (Pgp) and multidrug resistance-associated proteins (MRPs) that pump drugs out of a cell (Zaman et al, 1995; Hayes and Pulford, 1995). The glutathione/glutathione S-transferase detoxification system is frequently activated in MDR cells contributing to drug resistance (Altan et al, 1998). MRP acts in concert with this system, providing for the efflux of glutathione conjugates of xenobiotics from the cells (Zaman et al, 1995). Another impediment to treatment, which is present in MDR cells, involves the sequestration of drugs within cytoplasmic vesicles, followed by extrusion of the drug from the cell (Altan et al, 1998). Drug sequestration in MDR cells is achieved through the maintenance of abnormally elevated pH gradients across organelle membranes by the activity of $H^+$-ATPase, an ATP-dependent pump (Benderra et al, 1998). Energy-dependent processes associated with the above drug resistance mechanisms impose higher energy requirements upon the MDR cells, which might render these cells more sensitive to energy-depleting agents as opposed to the wild type cells, which have lower energy requirements (Kaplan et al, 1990). This paper, for the first time, demonstrates that Pluronic P85 (P85) induces a dramatic reduction in ATP levels selectively in MDR cells, while non-MDR cells are not responsive to this block copolymer in this manner. Energy depletion induced by P85 correlates with its observed sensitization effect in MDR cells with respect to doxorubicin. This provides a pivotal new insight regarding the mechanism of potentiation of cytotoxicity of select drugs by Pluronic block copolymers in MDR cells.

*Received 14 December 2000*
*Revised 25 September 2001*
*Accepted 27 September 2001*

*Correspondence to:* AV Kabanov

### MATERIALS AND METHODS

### Drugs

Doxorubicin was purchased from Sigma Chemical Co. (St Louis, MO, USA) and P85 (lot No. WPOP-587A) was provided by BASF Corp. (Parispany, NJ, USA). Doxorubicin/P85 was prepared by adding doxorubicin to the culture medium containing various concentrations of P85.

### Cell culture

The human epithelial cell line KB (ATCC CCL-17) and its MDR subline, KBv, derived by selection with vinblastine, were kindly provided by Dr DW Miller (University of Nebraska Medical Center, Omaha, NE, USA). These cells were cultured in DMEM with 10% FBS, 10 mM HEPES and penicillin/streptomycin; KBv cultures were supplemented with 1 µg/ml vinblastine (Faulding

Pharmaceutical Co, Elizabeth, NJ, USA). The human colon epithelium cell line, Caco-2, was kindly provided by RT Borchardt (University of Kansas, Lawrence, KS, USA) and was cultured in the media mentioned earlier. The murine myoblast line, C2C12 (ATCC CRL-1772), was kindly provided by P Lemieux (Supratek Pharma Inc., Montreal, PQ, Canada) and was cultured in the media mentioned earlier. The human breast carcinoma cell line MCF-7 (ATCC HT-B22) and its MDR subline MCF-7/ADR, derived by selection with doxorubicin, were kindly presented by YL Lee (William Beaumont Hospital, Royal Oak, Ml, USA). These cells were cultured in the same media mentioned earlier, except HEPES was increased to 1%, MCF-7/ADR cultures were supplemented with 10 ng/ml doxorubicin (Gensia Laboratories LTD, Irvine, CA, USA). The porcine kidney epithelial cell line, LLC-PK1 (ATCC CL-101) and its MDR subline, LLC-MDR1, derived by transfection with human MDR1 in the laboratory of Dr Piet Borst (The Netherlands Cancer Institute, Amsterdam, Netherlands), were obtained from Dr Erin Schuetz (St Jude Hospital, Memphis, TN, USA). These cells were cultured in Medium 199 with 10% FBS, 10 mM HEPES and penicillin/streptomycin; LLC-MDR1 cultures were supplemented with 640 nmol/ml vincristine (SP Pharmaceuticals Inc, Albuquerque, NM, USA). Human umbilical vein endothelial cells (HUVECs) were purchased from BioWhittaker (San Diego, CA, USA) and cultured in EGM-2 media (BioWhittaker). Bovine brain microvessel endothelial cells (BBMECs) were isolated and cultured as described previously (Fontaine, 1996). All other tissue culture reagents were obtained from Gibco Life Technologies, Inc. (Grand Island, NY, USA). The cells were seeded at a density of 25 000 cells/cm$^2$ into 24-well plates and were used for accumulation studies after reaching confluency (typically within 6–7 days).

### Western blot analysis

Identification of Pgp was done using the immunoblot technique described previously (Miller et al, 1996). The monoclonal antibodies used for Pgp were C219 (Dako Corp., Carpinteria, CA, USA). They were used at a dilution of 1:100. The secondary horseradish peroxide anti-mouse Ig antibodies (dilution 1:1500) were purchased from Amersham Life Sciences (Cleveland, OH, USA). The specific protein bands were visualized using a chemiluminescence kit (Pierce, Rockford, IL, USA).

### Determination of intracellular ATP

Cell monolayers were grown in 24-well plates until confluent. On the day of treatment, the medium was removed and replaced with assay buffer, which is composed of the following: NaCl (122 mM), $NaHCO_3$ (25 mM), glucose (10 mM), KCl (3 mM), $MgSO_4$ (1.2 mM), $K_2HPO_4$ (0.4 mM), $CaCl_2$ (1.4 mM) and HEPES (10 mM). Cells were equilibrated in this buffer for 30 min, then the assay buffer was removed and replaced with P85 treatment solutions (0.0001–5% wt.) in assay buffer. Cells were exposed to P85 for 2 h then washed twice with ice-cold PBS and solubilized in a 1% solution of Triton X-100 in PBS. The lysates were collected and frozen immediately (–20˚C) for subsequent ATP quantification (conducted within 24 h following the sample collection). ATP was determined as previously described using a luciferin/luciferase assay (Garewal et al, 1986). For this purpose, 100 µL aliquots of lysate were mixed with 100 µL of ATP assay mix (# FL-AAM, Sigma). Light emission was measured with a Turner Designs luminometer (model 20/20). Data were acquired as relative light units integrated over 20 s for samples and for standard calibration curves obtained using ATP standard (# FL-AAS, Sigma). ATP levels were normalized for protein content, which was assayed by using the Pierce BCA assay kit. Each data point represented the mean + SEM of a minimum of 4 replicates.

### Cytotoxicity assay

Cells were seeded in 96-well plates at a density of 5000 cells per well and allowed to attach overnight. Cells were exposed to doxorubicin alone or doxorubicin/P85 solutions (from 0.0001–5% P85) for 2 h at 37˚C, 5% $CO_2$. Cells were washed three times with the fresh medium and cultured for 3 d in the medium in the absence of the drug and P85. The cytotoxic activity of doxorubicin was then evaluated using a standard MTT assay (Ferrari et al, 1990). The absorbency at $\lambda$ = 450 nm was determined using a microKinetics Reader BT 2000. Each concentration point was repeated in eight wells. SEM values were less than 10%.

### Rhodamine 123 (R123) accumulation

R123 accumulation in cells was studied as previously described (Batrakova et al, 1999).

### Pgp ATPase assay

Membranes, expressing human Pgp (Gentest Co., MA, USA) were used to evaluate the effects of P85 on Pgp ATPase activity. A 0.06 ml reaction mixture containing 40 µg membranes, 20 µl assay buffer, with or 0.1% P85, were added to buffer solutions, containing 3–5 mM MgATP, 50 mM Tris-MES, 2 mM EGTA, 50 mM KCl, 2 mM dithiothreitol, and 5 mM sodium azide, and incubated at 37˚C for 20 min. An identical reaction mixture containing 100 µM sodium orthovanadate was assayed in parallel. The reactions were stopped by the addition of 30 µl of 10% SDS with antifoam A. Aliquots (200 µl) of ammonium molybdate in 15 mM zinc acetate: 10% ascorbic acid (1:4) were added to each sample and incubated for an additional 20 min at 37˚C. The liberation of inorganic phosphate was detected by its absorbance at 630 nm and quantitated by comparing the absorbance to a phosphate standard curve (Druekes et al, 1995).

### RESULTS

### Concentration dependency of P85 effects on ATP levels in MDR and non-MDR cells

To examine the effects of P85 on intracellular ATP levels, resistant (MCF-7/ADR) and sensitive (MCF-7) cells were exposed to this block copolymer at various concentrations (without drug) for 2 h. After that, cells were lysed and ATP was quantified. Figure 1A presents the ATP levels observed in resistant and sensitive cells following the treatment with P85. In the resistant cells, exposure to low concentrations of Pluronic (ca. 0.05% wt.) resulted in a decrease in the ATP level to 3.8% of the initial value. In contrast, in the sensitive cells, ATP levels did not drop until much higher concentrations of the block copolymer (ca. 5% wt.) were reached. At these P85 concentrations, the ATP levels in the sensitive cells decreased to 15% of the initial value. Therefore, exposure to low concentrations of P85 causes a selective decrease in ATP levels (energy depletion) in MDR cells but not in the sensitive cells.

 *© 2001 Cancer Research Campaign*

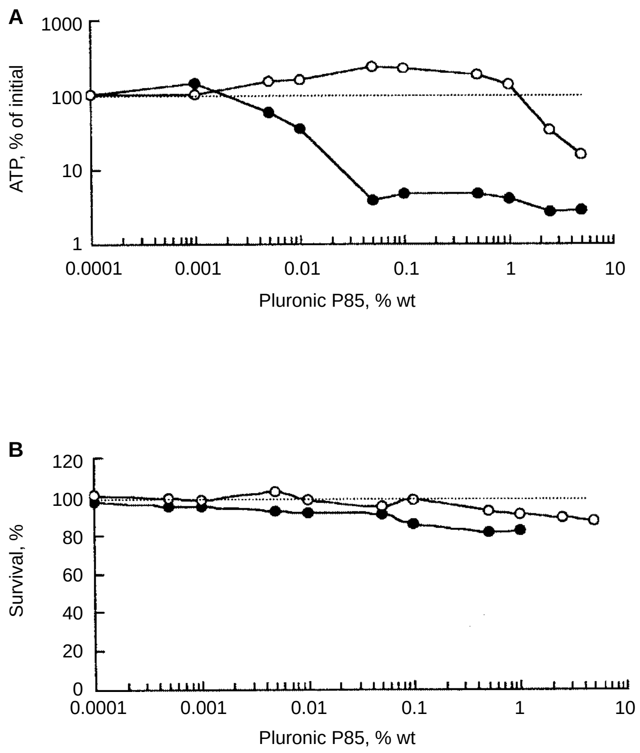

**Figure 1** Effects of P85 on (**A**) intracellular ATP levels; and (**B**) cell survival in resistant MCF-7/ADR (filled circles) and sensitive MCF-7 (empty circles) cells

### Kinetics of energy depletion

To determine the kinetics of P85-induced energy depletion, the MCF-7/ADR cells were exposed to the block copolymer for various time intervals and the ATP levels were determined as described in the previous section. The data are presented in Table 1. Rapid energy depletion was observed during first 15–30 min of cell exposure to P85. After that, ATP levels remained low and only slightly decreased during 2-hour exposure to the block copolymer. Similar time dependence of ATP depletion was observed in sensitive MCF-7 cells exposed to 5% P85 (a dose that causes energy depletion in these cells).

### Energy restoration following P85 exposure

The ability of the cells to restore ATP levels following removal of P85 from the culture medium was tested in the following experiment. MCF-7/ADR cells were exposed to 0.1% P85 for 2 h, to induce reduced ATP levels, then the P85-containing supernatants were removed and cells were cultured in the copolymer-free media for various time periods. Measurements of the ATP levels at various timepoints after removal of P85 suggested that ATP levels restored after 15 h, and then maintained at the pre-treatment levels for at least 70 h (Table 2). In a similar experiment, sensitive MCF-7 cells were exposed to 5% P85 and then cultured in the absence of P85 for various time periods to examine energy restoration. In this case, ATP levels were also restored after the 15-hour interval (Table 2).

### Absence of cytotoxic effects of P85

To examine whether exposure to Pluronic affects the viability of cells, both resistant and sensitive cells were exposed to various doses of P85 (without drug) for 2 h. After removal of the P85, cells were cultured in the block-copolymer free media for 72 h and cytotoxicity was determined using MTT assay. As in seen in Figure 1, 2-hour exposure to P85 did not induce significant cytotoxic effects in either resistant or sensitive cells over the range of concentrations tested (from 0.0001 to 5%).

**Table 1** Kinetics of ATP depletion in MCF-7/ADR cells in the presence of 0.1% P85

| Time of exposure (min) | ATP[a], nmol/mg protein |
|---|---|
| 0 | 300 + 32 |
| 15 | 61.4 + 4.6 |
| 30 | 40.2 + 7.2 |
| 60 | 33.4 + 11 |
| 90 | 26.2 + 0.9 |
| 120 | 25.5 + 0.6 |

[a]Mean + SEM (*n* = 4).

**Table 2** Intracellular ATP levels before and after treatment of MCF-7/ADR or MCF-7 cells with P85[a]

| Time of measurement of ATP level | ATP, nmol/mg protein[b] | |
|---|---|---|
| | MCF-7/ADR | MCF-7 |
| Before P85 treatment | 302 + 21 | 29.0 + 1.4 |
| Immediately after 2 h exposure to P85 | 15.3 + 2.3 | 2.4 + 0.34 |
| After P85 removal: | | |
| 4 h | 21.2 + 2.7 | 14.9 + 1.5 |
| 15 h | 402 + 13 | 40.0 + 1.9 |
| 39 h | 325 + 31 | 22.3 + 1.1 |
| 48 h | 265 + 52 | 27.6 + 2.4 |
| 70 h | 301 + 28 | 28.5 + 1.5 |

[a]Cells were exposed to 0.1% (MCF-7/ADR) or 5% (MCF-7) P85 and then incubated for various time periods in the copolymer-free culture media.
[b]Mean + SEM (*n* = 4).

### Responsiveness of various cells to P85-induced energy depletion

To examine the correlation of the P85-induced energy depletion with the expression of the drug efflux transporters in the cells, this study compared the effects of the block copolymer on ATP levels in several cell types that either express Pgp, or do not express Pgp. First, the cell panel used in this study included pairs of resistant and sensitive cancer cell lines: MCF-7/ADR and MCF-7 as well as KBv and KB. Second, the panel included cells with intrinsic Pgp expression, Caco-2 and BBMECs, as well as Pgp-negative cells, human umbilical vein endothelial cells (HUVECs) and C2C12 myoblasts. Finally, this panel included porcine kidney cells, LLC-MDR1, stably expressing the MDR1 gene, and its counterpart, LLC-PK1, with low endogenous Pgp levels (Evers et al, 1996). The levels of expression of Pgp in the cells used in this study were confirmed by a Western blot analysis (Figure 2). In this study the drug-selected MDR and MDR1-transfected lines (MCF-7/ADR, KBv, LLC-MDR1) exhibited very high levels of Pgp. Pgp was also present in BBMECs and Caco-2 cells, although its amounts were much lower than in drug-selected and -transfected MDR cells. All sensitive cell lines revealed much lower levels of endogenous Pgp compared to the MDR cells. The functional activity of Pgp was validated using R123 as a Pgp-sensitive probe (Lee et al, 1994).

© 2001 Cancer Research Campaign

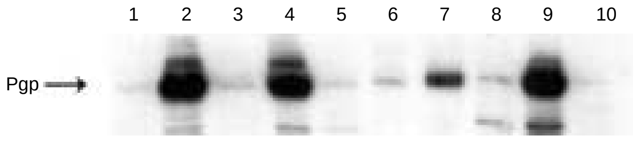

**Figure 2** Western blot analysis of Pgp in various cell lines: (1) MCF-7, (2) MCF-7/ADR, (3) Kb, (4) Kbv, (5) HUVECs, (6) Caco-2, (7) BBMECs, (8) LLC-PK1, (9) LLC-MDR1, (10) C2C12. A total of 25 µg of protein was loaded onto 7.5% polyacrylamide gels. Primary antibodies for Pgp (C219) were used at dilution of 1:100; secondary antibody was used at a dilution of 1:1500. The protein was detected using chemiluminescence

In the energy depletion study, the cells were exposed for 2 h to various concentrations of P85 and then ATP levels were determined as described earlier. The effective concentrations of P85 that induced a 50% decrease in ATP levels in the cells ($EC_{50}$), as determined from the dose-response curves are presented in Table 3. This table also presents the relative responsiveness of the Pgp-expressing cells compared to their counterparts with low levels of Pgp expression. While there were significant differences in the initial ATP levels observed between various cell lines and these differences were very well reproduced in repeated experiments (not shown in Table 3), the responsiveness of the cells to P85 did not appear to correlate with the amount of ATP available in these cells. Conversely, as is seen in Table 3, the responsiveness of the cells to Pluronic correlated with expression of Pgp in these cells. Specifically, energy depletion was observed in the resistant cell lines MCF-7/ADR and KBv, but not in the sensitive parental cells lines MCF-7 and KB. Likewise, Caco-2 and BBMECs, which have functionally active Pgp (Batrakova et al, 1998), responded to P85 at lower concentrations of the block copolymer than the HUVECs and C2C12 cells, which have low levels of Pgp expression by Western blot and functional assays. Finally, transfected LLC-MDR1 cells, overexpressing Pgp, were highly responsive to P85 treatment, while their non-Pgp counterparts were much less responsive to such treatment. Based on the results of this study, it appears that the expression of drug efflux proteins is one factor that renders cellular metabolism responsive to treatment with P85. Among cells with the lowest level of Pgp expression, the highest responsiveness to P85 treatment was observed for HUVECs which exhibited an $EC_{50}$ value of 0.0675% (Table 3). Although these cell lines were characterized as Pgp negative (Iwahana et al, 1998), it might express some other transport systems that could play a similar role as Pgp, resulting in the response to the block copolymer. Furthermore, Caco-2 cells, which exhibited a relatively small amount of Pgp in the Western blot analysis compared to the drug-selected and MDR1-transfected cells, were very responsive to P85 in the energy depletion study. Once again Caco-2 cells may have some other factors present, besides the Pgp efflux system, which determine their high responsiveness to the block copolymer.

### Selective energy depletion by metabolic inhibitors

To compare the effects of P85 and known metabolic inhibitors, MCF-7/ADR and MCF-7 cells were exposed for 2 h to various inhibitors, rotenone, sodium aside, and 2-deoxyglucose as well as P85. After that the intracellular ATP levels were measured and the $EC_{50}$ values were determined from the dose-response curves as described in the previous section. Table 4 shows the results of this experiment. All metabolic inhibitors examined appeared to induce selective energy depletion in MDR cells when compared to non-MDR cells. The selectivity of these agents with respect to MDR cells varied from about 10 to about 200 times (as determined by the values of the relative responsiveness presented in the table).

### Relationship between energy depletion and cytotoxicity

The effect of P85 on the cytotoxicity of doxorubicin was determined using drug-selected and MDR1-transfected, resistant cells (MCF-7/ADR, KBv, LLC-MDR1) as well as their non-MDR counterparts (MCF-7, KB, LLC-PK1). The resulting $IC_{50}$ values of doxorubicin determined in these cell lines without and with 0.1% P85 are presented in Table 5. Comparison of these data with the ATP depletion studies presented in Table 3, provides initial

**Table 4** Comparison of the effects of P85 and metabolic inhibitors on ATP levels in MDR and non-MDR cells

| Agent | $EC_{50}$,[a] µM | | Relative responsiveness[b] |
|---|---|---|---|
| | MCF-7 | MCF-7/ADR | |
| P85 | 5000 | 20 | 250 |
| Rotenone | 0.03 | 0.0008 | 37 |
| Sodium aside | 100 000 | 10 000 | 10 |
| 2-deoxyglucose | 200 | 1 | 200 |

[a]Mean + SEM ($n$ = 4). [b]Calculated as the ratio of $EC_{50}$ of MCF-7 cells to $EC_{50}$ of MCF-7/ADR cells.

**Table 3** Effects of P85 on ATP levels in various cells following 2-hour exposure to the block copolymer

| Cells | Pgp or MRP overexpression | Initial ATP levels nmol/mg protein[a] | $EC_{50}$ (%) | Relative responsiveness to P85[b] |
|---|---|---|---|---|
| MCF-7 | No | 30 + 1.5 | 2.25 | – |
| MCF-7/ADR | Pgp | 300 + 20 | 0.009 | 250[c] |
| KB | No | 1 + 0.01 | 0.675 | – |
| KBv | Pgp | 4 + 0.1 | 0.036 | 19[d] |
| C2C12 | No | 15 + 1.4 | 4.5 | – |
| HUVECs | No | 40 + 4.9 | 0.0675 | – |
| Caco-2 | Pgp | 5.5 + 0.4 | 0.00067 | 6670[e] |
| BBMECs | Pgp | 1.6 + 0.04 | 0.018 | 250[d] |
| LLC-PK1 | No | 61 + 6.9 | 0.45 | – |
| LLC-MDR1 | Pgp | 79 + 1.7 | 0.0045 | 100[f] |

[a]Mean + SEM ($n$ = 4). [b]Calculated as the ratio of $EC_{50}$ of non-Pgp cells to $EC_{50}$ of corresponding Pgp expressing cells. [c]Compared to MCF-7 cells. [d]Compared to KB cells. [e]Compared to C2C12 cells. [f]Compared to LLC-PK1 cells.

 *© 2001 Cancer Research Campaign*

**Table 5** Effects of P85 on $IC_{50}$ of doxorubicin in drug-selected and -transfected MDR cell lines as well as the non-MDR cells

| Cells | $IC_{50}$, μg/ml[a] | | Relative responsiveness to P85[b] |
|---|---|---|---|
| | Assay buffer | 0.1% P85 | |
| MCF-7/ADR | 900 ± 5 | 4.5 ± 0.2 | 200 |
| MCF-7 | 2.0 ± 0.1 | 2.0 ± 0.1 | 1 |
| KBv | 5300 ± 21 | 12.6 ± 0.1 | 420 |
| KB | 1.00 ± 0.03 | 1.00 ± 0.03 | 1 |
| LLC-MDR1 | 200 ± 11 | 0.80 ± 0.04 | 250 |
| LLC-PK1 | 2.0 ± 0.1 | 2.0 ± 0.1 | 1 |

[a]Mean ± SEM ($n$ = 4). [b]Calculated as the ratio of $IC_{50}$ of control non-treated cell to $IC_{50}$ of treated with most effective concentration of P85.

evidence of the relationship between the sensitization effect and the energy depletion phenomenon.

This relationship is further illustrated by the dose-dependent studies shown in Figure 3. Figure 3A presents the $IC_{50}$ in the resistant cells, KBv upon co-administration of doxorubicin with various doses of P85. The data show that P85 potentiates the cytotoxic activity of doxorubicin with respect to MDR cells ($IC_{50}$ decreases). This effect was registered at approximately the same doses of P85 that cause energy depletion in MDR cells (also shown in Figure 3A). In contrast, treatment with the same or higher doses of P85 (up to at least 1%) did not result in a similar potentiation of cytotoxicity in the non-MDR line, KB (data not shown).

Figure 3B summarizes the data of Figure 3A on the doxorubicin cytotoxicity and energy depletion studies in resistant KBv cells. This presents the relationship between (1) the ATP levels determined after exposure of the cells to various concentrations of P85 and (2) the $IC_{50}$ of doxorubicin determined in the presence of the same concentrations of P85. These studies suggest that the effect of P85 on the ATP levels in MDR cells correlates well with the sensitization effect of this block copolymer in MDR cells. The lower the ATP level is, the more cytotoxic the drug becomes. Very similar results were obtained using another resistant cell line, MCF-7/ADR (data not shown).

### Energy supplementation abolishes sensitization of resistant cells by P85

The relationship between P85-induced changes in ATP levels and the sensitization effect in the resistant cells was further examined using KBv cells. This cell line was exposed for 2 h to doxorubicin alone or doxorubicin formulated with 0.1% P85. In an attempt to bypass Pluronic-induced energy depletion, one more treatment group was also included in this study, in which doxorubicin/P85 was supplemented with 50 μM ATP and $10^{-5}$ M dodecylamine, as a permeabilizing agent. As reported previously by Slepnev et al (1992), treatment of the cells with dodecylamine in combination with P85 allows transport of ATP into the cells from the extracellular media. The role of dodecylamine in this system is to provide for the positive charge that enhances binding of the ATP molecule with the block copolymer aggregates. These aggregates containing adsorbed ATP subsequently are internalized within the cells (Slepnev et al, 1992). Following exposure of the cells to doxorubicin solutions, drug-induced cytotoxicity was determined. In a parallel study, the KBv cells were exposed for 2 h to the same treatment solutions, without doxorubicin, and intracellular ATP levels were determined. As is seen in Figure 4A, in the absence of ATP in the extracellular media, P85 induced a substantial decrease in ATP in KBv cells. The cytotoxic activity of doxorubicin was also enhanced in this case, resulting in ca. 83-fold decrease in $IC_{50}$ for the doxorubicin/P85 formulation compared to the $IC_{50}$ of the free drug. However, when the external media was supplemented with ATP and dodecylamine, both the energy depletion and sensitization effects of P85 were considerably reduced. In this case, the $IC_{50}$ value of doxorubicin formulated with Pluronic was only 5.5 times less than the $IC_{50}$ of free doxorubicin. No effect from the addition of extracellular ATP and dodecylamine on the $IC_{50}$ of the drug was observed in the absence of P85 (data not shown). To validate the results of this study using a drug-selected MDR cell line, KBv, the same experiment was repeated with the MDR1-transfected cell line, LLC-MDR1. The result was essentially the same (Figure 4B). P85 sensitized the cells, decreasing the $IC_{50}$ of the drug by over 100-fold. The intracellular ATP levels were also decreased as already discussed. The treatment of the cells with the energy supplementation system in this case

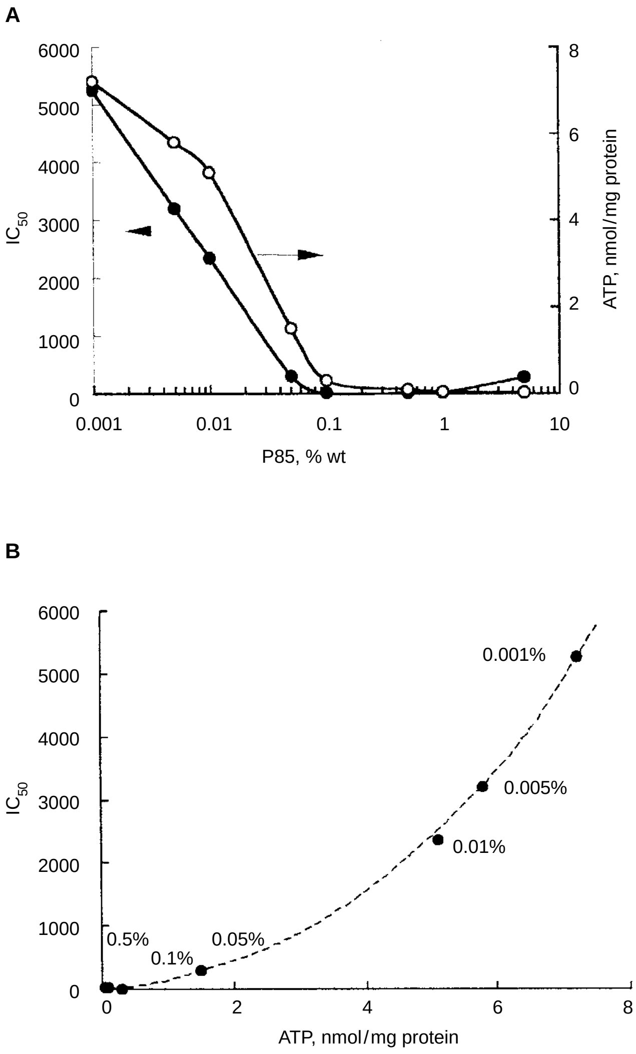

**Figure 3** (**A**) Effects of P85 on $IC_{50}$ of doxorubicin (filled symbols) and ATP intracellular levels (empty symbols) in KBv cells. (**B**) Relationship between the P85-induced changes in $IC_{50}$ of doxorubicin and ATP levels in KBv cells. Cells were exposed to treatment solutions for 2 h

*© 2001 Cancer Research Campaign* 

resulted in the intracellular ATP levels that exceeded the initial values. The increase in the ATP levels in the presence of the energy supplementation system was accompanied by practically complete abolishment of the sensitization effect of the block copolymer. Overall, based on these studies, it appears that there is a direct relationship between the levels of ATP and sensitization of resistant cells with respect to doxorubicin by P85.

### Energy depletion by metabolic inhibitor sensitizes resistant cells

This relationship was further validated by comparing the effect of P85 with that of the combination of metabolic inhibitors, 2-deoxyglucose and sodium azide, in KBv and LLC-MDR1 cells. For this purpose, additional treatment groups were included in the studies discussed in the previous section. In these groups the cells were treated by exposure to a mixture of 2-deoxyglucose (50 mM) and sodium azide (150 µM). The intracellular ATP levels and cytotoxicity of doxorubicin following this treatment were determined as described earlier. As is seen in the figures, exposure of both cell lines, KBv (Figure 4A) and LLC-MDR1 (Figure 4B) to the 2-deoxyglucose and sodium azide mixture resulted in a considerable decrease in ATP, which was comparable in magnitude to the effect of P85 observed in these cells. Furthermore, the decrease in ATP levels induced by the metabolic inhibitors was also accompanied by an increase in the sensitivity of the cells to the drug. It is noteworthy that in the KBv cells, the effect of the metabolic inhibitor on the cytotoxicity of doxorubicin has the same magnitude as the sensitization effect of P85 in these cells (Figure 4A). Conversely, in LLC-MDR1 (Figure 4B), the metabolic inhibitors appeared to be much less potent than the block copolymer and induced only a 10-fold decrease in $IC_{50}$ of doxorubicin compared to over a 100-fold effect of P85 in these cells. Overall, the studies reported in this section suggest that inhibition of metabolism in the resistant cells leads to the sensitization of the cells with respect to antineoplastic agent cytotoxic action.

### Comparison of different Pluronic copolymers

Figure 5 presents the effects of two Pluronic copolymers, L61 and F127 on the intracellular ATP levels in the MDR cell line MCF-7/ADR (panel A) and LLC-MDR1 (panel B). Both copolymers consist of ethylene oxide (EO) and propylene oxide (PO) segments arranged in a basic structure: $EO_a$-$PO_b$-$EO_a$, but they differ in the overall lengths and weight proportion of these segments. Previous study demonstrated that L61 is a potent sensitizing agent for MDR cells, while F127 is inactive in this respect (Batrakova et al, 1999). As seen in Figure 5, exposure of both cell lines to low concentrations of L61 (ca. 0.01% wt.) resulted in a decrease in ATP levels. In contrast, in the F127 treatment groups, ATP levels did not decrease until very high concentrations of the block copolymer (10% wt.) were reached. This provides further evidence supporting the relationship between the ability of Pluronic block copolymers to decrease ATP levels and sensitize MDR cells.

### Relationship between Pgp inhibition and energy depletion

The fluorescent dye, R123 was used in this work to examine the functional activity of the Pgp efflux pump (Lee et al, 1994). Figure 6 presents the accumulation of R123 in the Pgp

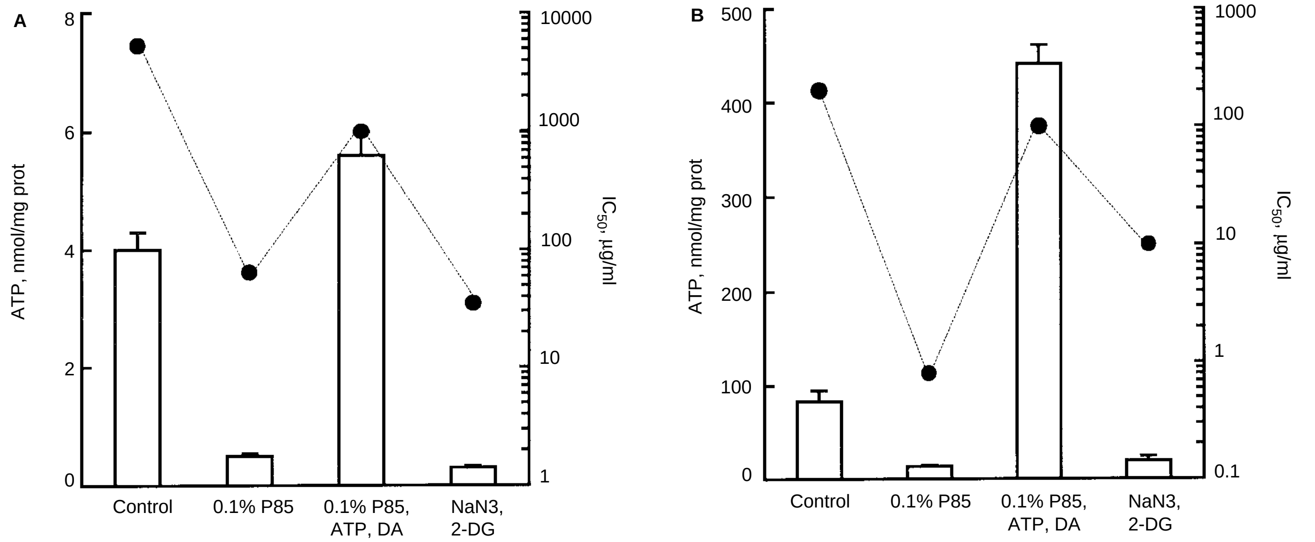

**Figure 4** Intracellular ATP levels (empty bars) and doxorubicin cytotoxicity ($IC_{50}$, filled dots) modulated by various agents in (**A**) KBv cells and (**B**) LLC-MDR1 cells. ATP levels (means + SEM, *n* = 4) were determined after exposure of the cells for 2 h to the following treatment solutions: (1) copolymer-free media, (2) P85 (0.1%), (3) P85 (0.1%) in the presence of extracellular ATP (50 µM) and dodecylamine ($10^{-5}$ M), (4) sodium azide (150 µM) and 2-deoxyglucose (50 mM). For cytotoxicity studies, the corresponding treatment solutions also contained various concentrations of doxorubicin, and $IC_{50}$ values were determined using a standard MTT assay following 3-day culturing of the cells in the drug-free medium

 *© 2001 Cancer Research Campaign*

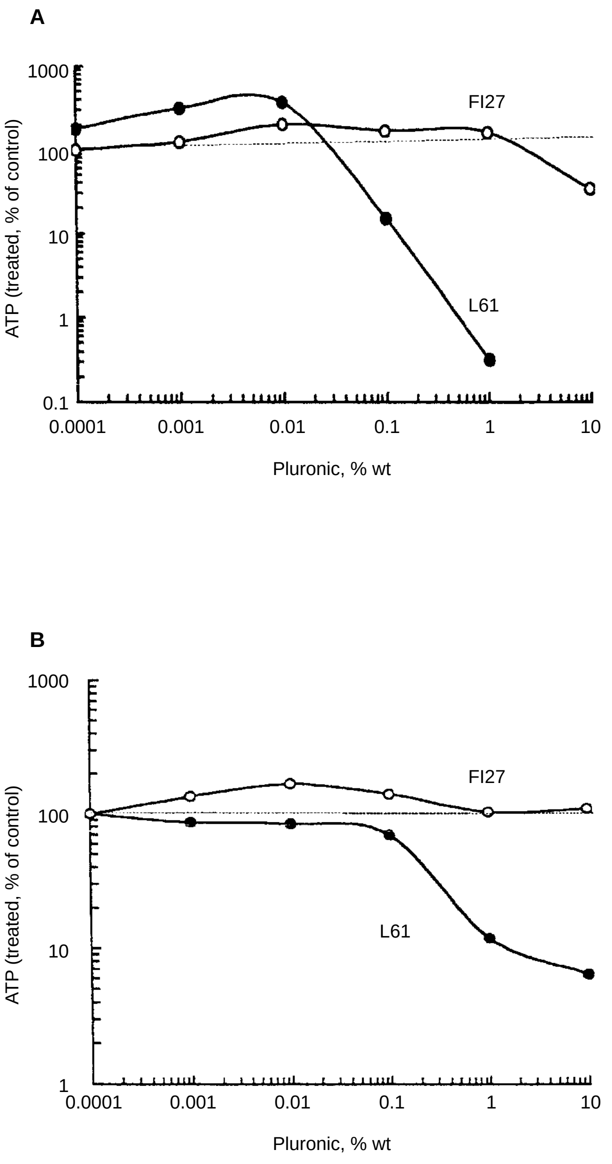

**Figure 5** Intracellular ATP levels in (**A**) MCF-7/ADR cells and (**B**) LLC-MDR1 cells measured following 2-hour exposure of cells to various concentrations of Pluronic L61 (filled circles) or F127 (empty circles). The dashed line represents the ATP level (100%) in the media controls overexpressing cells, KBv in the presence of various doses of P85. The data show that at lower concentrations of from 0.001–0.1%, P85 increases the accumulation of the probe in the cells. According to the previous studies, this effect is due to the inhibition of the Pgp efflux system (Batrakova et al, 1999; Evers et al, 2000). At higher concentrations of P85 (0.1–5%) the R123 levels decrease. The effect of high P85 concentrations is believed to be due to incorporation of the probe in the P85 micelles, resulting in the decrease in the amounts of the free probe available for diffusion into the cells (Batrakova et al, 1999). Figure 6 also presents the data on the P85-induced energy depletion in MCF-7/ADR cells. It is seen from this data that at lower concentrations of P85 (0.001–0.1% P85), when the transport of the probe is not impeded by its incorporation in the P85 micelles, rhodamine accumulations change inversely to the ATP changes.

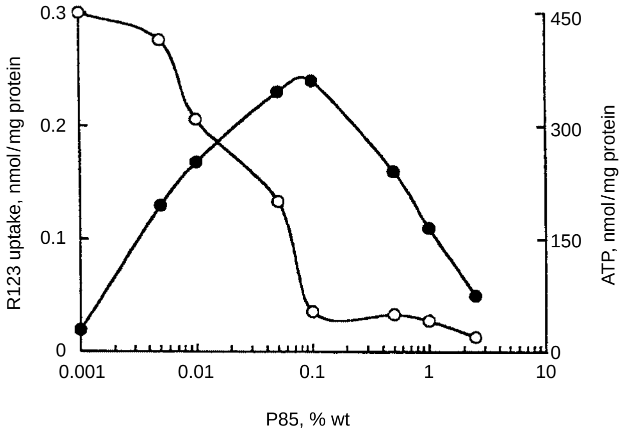

**Figure 6** Relationship between the P85-induced changes in R123 accumulation (filled circles) and ATP levels (empty circles) in MCF-7/ADR cells. Cells were exposed to treatment solutions for 1.5 h

This suggests that the lower the ATP level is, the less active the Pgp efflux system becomes. The same results were obtained using the KBv cell line.

### Energy supplementation activates Pgp efflux

The following study evaluated whether energy supplementation can restore the Pgp efflux function in the resistant cells in the presence of P85. The experiment was conducted using MCF-7/ADR cells analogously to the energy supplementation described earlier. Rhodamine accumulation in MCF-7/ADR cells was examined for five treatment groups:

1. The probe in the assay buffer
2. The probe in 0.1% P85 solution
3. The probe in 0.1% P85 solution supplemented with a mixture of 50 μM ATP and $10^{-5}$ M dodecylamine
4. The probe in 0.1% P85 solution and $10^{-5}$ M dodecylamine
5. The probe in $10^{-5}$ M dodecylamine.

In a parallel study, intracellular ATP levels were also determined following exposure of the MCF-7/ADR cells to the corresponding treatment solutions (without probe). As is seen in Figure 7A, there was an inverse correlation between the R123 uptake and ATP intracellular levels. First, in the presence of P85, the ATP level was decreased while the probe accumulation was increased compared to the assay buffer controls. Second, when the P85 treatment was supplemented by the energy regeneration system that elevated the intracellular ATP level, the rhodamine uptake was drastically decreased, which indicates that the function of Pgp was restored. This treatment group indicates even lower R123 uptake than that observed in the assay buffer control (significant, $P < 0.05$). At the same time, it is noteworthy that the energy regeneration system produced higher ATP intracellular levels than the levels observed in the control cells. Two last treatment groups were included in the study as the controls. First, dodecylamine alone without P85 added does not change R123 accumulation or ATP intracellular levels in MCF-7/ADR cells. Second, the mixture of the dodecylamine and P85 without extracellular ATP added exhibits exactly the same effect on R123 accumulation in the cells or ATP intracellular levels as the block copolymer alone. These controls exclude the possibility that dodecylamine alone or dodecylamine in

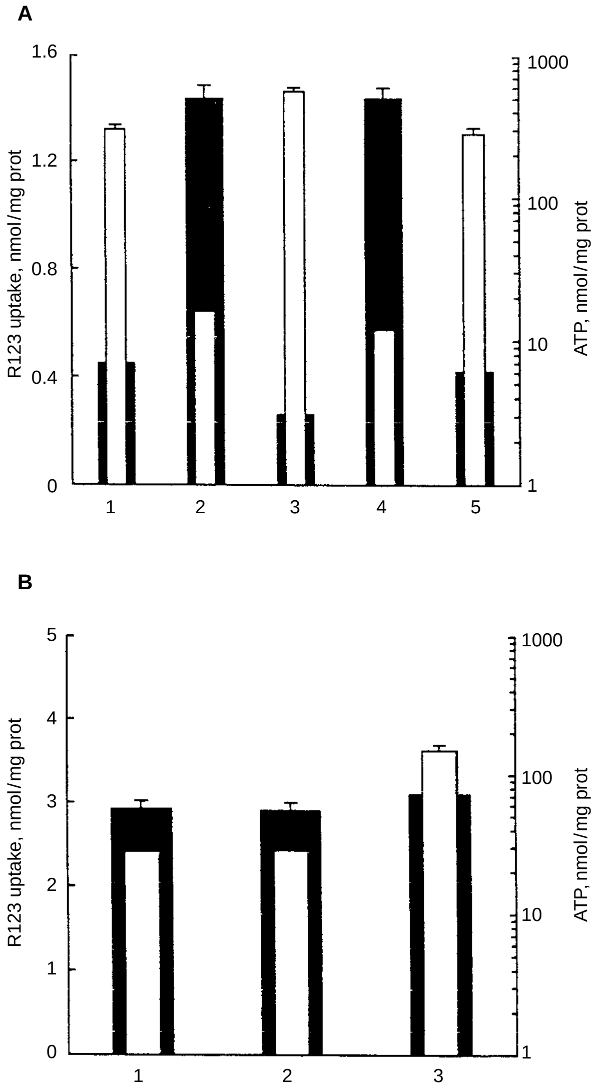

**Figure 7** Intracellular ATP levels (empty bars) and R123 accumulation (filled bars) modulated by various agents in (**A**) MCF-7/ADR cells and (**B**) MCF-7 cells. ATP levels (means + SEM, $n$ = 4) were determined after exposure of the cells for 1.5 h to the following treatment solutions: (1) copolymer-free media, (2) P85 (0.1%), (3) P85 (0.1%) in the presence of extracellular ATP (50 μM) and dodecylamine ($10^{-5}$ M), (4) P85 (0.1%) and dodecylamine ($10^{-5}$ M) and (5) dodecylamine ($10^{-5}$ M). For R123 accumulation studies the same treatment solutions also contained 3.2 μM of the probe.

combination with P85 could be inhibitors of the drug efflux system. Furthermore, these results suggest that in the case when the energy supplementation system was used, the observed restoration of Pgp efflux is due to the presence of ATP rather than some non-specific perturbations in the membrane structure caused by dodecylamine.

In another study, the effects of the energy supplementation system on R123 accumulation and ATP intracellular levels was evaluated using the sensitive MCF-7 cell line, which has low levels of the endogenous Pgp as confirmed in the prior study using the Western blot analysis (Figure 2). As is seen in Figure 7A exposure of these cells to 0.1% P85 did not affect either R123 accumulation or intracellular ATP levels. When these cells were exposed to the energy supplementation system, the ATP levels were substantially increased. However, the transport of R123 in the sensitive MCF-7 cells remained unchanged. This study reinforces the conclusion that P85 and/or dodecylamine have no effect on permeability of the cell membrane in non-MDR cells. Furthermore, the increased absorption of the R123 probe in the MDR cells is attributable to the effects of the block copolymer on the Pgp efflux system, rather than to non-specific alterations in membrane permeability of the probe.

The final experiment reported in this section examined whether energy supplementation could affect the Pgp efflux system in the MDR1-transfected cell line, LLC-MDR1. As is seen from the data presented in Table 6, the effect of the energy supplementation system in these cells is very similar to the effect of this system in MCF-7ADR cells described earlier. Indeed, inhibition of Pgp is observed in these cells following their exposure to 0.1% P85. However, in the presence of the dodecylamine and extracellular ATP, the Pgp efflux is restored. No changes in the R123 accumulation were observed when cells were treated with the dodecylamine alone, which once again confirms that there were no inhibitory effects of dodecylamine on MDR1.

### Effect of P85 on Pgp ATPase activity

The effects of P85 on the Pgp ATPase activity were evaluated using isolated membranes containing human Pgp by assaying the liberated inorganic phosphate (Druekes et al, 1995). The treatment solutions included the copolymer-free buffer control and solutions containing various concentrations of P85. An additional treatment group in which the Pgp substrate, verapamil, was added to either copolymer-free buffer or P85 solutions was also evaluated. The purpose of including the verapamil groups was to determine whether binding of a specific substrate with Pgp could modulate the effects of P85 on Pgp ATPase activity. As seen in Table 7, P85 induced dramatic decreases in Pgp ATPase activity compared to the copolymer-free control. This inhibitory effect was observed at concentrations of P85 as low as 0.001% as well as at the higher

**Table 6** Effects of P85, extracellular ATP and dodecylamine on R123 accumulation in monolayer of LLC-MDR1 cells

| Treatment groups | R123 accumulation (nmol/mg prot) |
|---|---|
| Assay buffer | 0.090 ± 0.003 |
| P85 (0.1%) | 0.32 ± 0.02 |
| 0.1% P85, 50 μM ATP and $10^{-5}$ M dodecylamine | 0.080 ± 0.001 |
| $10^{-5}$ M dodecylamine | 0.10 ± 0.01 |

**Table 7** Effect of P85 on Pgp ATPase activity in isolated membranes containing human Pgp

| Concentration of P85 (% wt.) | Pgp ATPase activity, nmol/mg prot × min[a] | |
|---|---|---|
| | In the absence of vinblastine | In the presence of vinblastine |
| 0 | 9.40 ± 0.40 | 19.50 ± 0.25 |
| 0.001 | 1.02 ± 0.05 | 3.21 ± 0.11 |
| 0.01 | 0.64 ± 0.02 | 1.76 ± 0.07 |
| 0.1 | 1.34 ± 0.05 | 0.32 ± 0.02 |
| 1 | 4.22 ± 0.15 | 3.42 ± 0.12 |

[a]Mean ± SEM ($n$ = 4).

 *© 2001 Cancer Research Campaign*

concentrations examined (up to 1%). Furthermore, the inhibitory effect of P85 was observed in the presence of verapamil at all block copolymer concentrations examined (0.001–1%). Verapamil alone in the absence of the block copolymer induced a significant increase in Pgp ATPase activity. This effect is believed to be due to the binding of verapamil in the active centre of Pgp (Shepard et al, 1998; Rebbeor and Senior, 1998). Furthermore, it is noteworthy that both in the presence and absence of verapamil, the Pgp ATPase activity was in part restored at 1% P85. In these treatment groups, the Pgp ATPase activity reached ca. 40–45% of that observed in the verapamil- and P85-free controls. Therefore, this study suggested that P85 significantly decreases in Pgp ATPase activity, overcoming the effect of verapamil activation of the Pgp efflux system.

### DISCUSSION

The key observation that Pluronic block copolymer reverse MDR was published previously (Alakhov et al, 1996; Venne et al, 1996), and that the Pluronic-based formulation of doxorubicin is undergoing clinical trial now. However the mechanisms of the effects of Pluronic copolymers in MDR cells not are completely understood. This paper provides key insight into these mechanisms and for the first time relates it to a selective energy depletion induced by Pluronic in resistant cells.

Exposure of resistant and sensitive cells to different doses of P85 resulted in a transient energy depletion, which was reversed when the block copolymer was removed. The resistant cells were much more responsive to P85, exhibiting profound decreases in ATP levels at 100 times' lower concentration of the block copolymer compared to the sensitive cells. The observed transient energy depletion, in the absence of antineoplastic agent, was not accompanied by observable toxicity in the cells since Pluronic alone did not exert any significant cytotoxic effect in either the resistant or the sensitive cells. However, if the antineoplastic agent, doxorubicin, was introduced concurrently with the block copolymer, the cytotoxic effect of doxorubicin was significantly increased compared to the drug alone.

Overall, based upon this study, the sensitization effect of the block copolymer in resistant cells appears to be related to energy depletion. First, there is a clear correlation between the potentiation of the cytotoxicity of doxorubicin and ATP depletion induced by the block copolymer observed in both drug-selected and transfected MDR cells. Second, ATP depletion by the metabolic inhibitors is also accompanied by the potentiation of the cytotoxic activity in the MDR cells. Finally, this work demonstrates that treatment of the cells with the energy-supplementation system reverses the sensitization of the MDR cells by Pluronic.

Previous work has demonstrated that Pluronic affects multiple mechanisms of drug resistance in MDR cells. There is overwhelming evidence to suggest that Pluronic block copolymers inhibit Pgp efflux systems in Pgp-expressing cells (Venne et al, 1996; Batrakova et al, 1998; 1999). No alteration in drug uptake in the presence of Pluronic was observed with non-MDR cells. This provides additional support for the specific effects of the copolymer on the Pgp transport system. This conclusion is consistent with the recent studies by Evers et al (2000) and Batrakova et al (2001), demonstrating that Pluronic block copolymers (L61, P85) have pronounced effects, increasing accumulation and permeability of various Pgp-dependent drugs in MDR1-transfected cells, which overexpress Pgp. In addition to the effects on the Pgp efflux system, Pluronic block copolymer can affect other drug-resistant mechanisms present in MDR cells. For example, a previous study by Venne et al (1996) demonstrated that exposure of the MDR cells to Pluronic block copolymers abolishes sequestration of drugs in the cytoplasmic compartments.

Both the drug-efflux systems and the drug-sequestration mechanisms in MDR cells are ATP dependent. This work suggests that there is a clear relationship between the energy depletion and Pgp inhibition induced by the block copolymer: the lower the ATP level, the less active the Pgp-efflux system becomes. Furthermore, treatment of the cells with the energy-supplementation system, which restores ATP levels within the cells, also reverses the effect of P85 on Pgp. It is tempting to suggest that the energy depletion is the sole reason for inhibition of various ATP-dependent drug resistance mechanisms in MDR cells. However, using isolated membranes expressing Pgp, this study also demonstrates that P85 can inhibit the Pgp ATPase activity. Therefore, the block copolymer may have a 2-fold effect in MDR cells – one through ATP depletion and another through inhibition of the Pgp ATPase activity; both have a combined result of potent inhibition of Pgp.

Previous reports have suggested that certain Pluronic block copolymers, including P85, studied in this work, can affect metabolism in cells. For example, decreased ATP levels were observed following exposure to P85, in the Jurkat T-cell lymphoma (Slepnev et al, 1992). Furthermore, a study by Kirillova et al (1993) suggested that P85 inhibits respiration, both in isolated mitochondria and in whole cells. One important observation made in the present work is that MDR cells are much more responsive to Pluronic than non-MDR cells. The reasons for such a remarkable selectivity of Pluronic block copolymers with respect to MDR cells are not completely understood. One hypothesis is that the high rates of energy consumption by the drug efflux pumps, combined with Pluronic-induced inhibition of respiration, determine the responsiveness of the resistant cells to the block copolymer. Under conditions in which the respiration necessary for ATP synthesis is inhibited, the high rates of ATPase activity by the drug efflux pumps (and possibly, some other energy-dependent mechanisms) could result in a rapid exhaustion of the intracellular ATP pools in the resistant cells. Alternatively, cells that do not exhibit these resistance mechanisms would appear to be less responsive to inhibition of respiration and would not exhibit energy depletion, at least to the extent observed in the resistant cells. Such a hypothesis is in line with the earlier observation that resistant cells have an increased glucose utilization rate compared to sensitive cells (Kaplan et al, 1991). Furthermore, the toxicity of 2-deoxyglucose, a glucose antimetabolite, was found to be consistently higher in MDR cells than in the parental drug-sensitive lines (Kaplan et al, 1990; 1991). This study directly demonstrates that metabolic inhibitors deplete ATP in the resistant cells more than in the sensitive cells. The extent of the selectivity of P85 with respect to MDR cells is the same of even greater than that of the inhibitor of glycolysis, 2-deoxyglucose. Furthermore, P85 is significantly more selective than the inhibitors of respiration, rotenone and sodium aside. However, the present study also suggested that P85 significantly inhibits ATPase activity of Pgp in isolated cell membranes. Since Pgp is one of the major ATPases overexpressed in MDR cells and the fact that Pluronic inhibits this activity, it makes the high energy consumption in MDR cells a less likely cause for the block copolymer selectivity in these cells. An alternative hypothesis would be that for some reason the metabolic processes in MDR cells are more sensitive to inhibition with Pluronic than the metabolic processes in non-MDR cells.

*© 2001 Cancer Research Campaign* 

This could result in the more pronounced ATP depletion observed following exposure of MDR cells to the block copolymer. It also has to be emphasized that overexpression of Pgp is not the only reason for the appearance of the MDR phenotype. The MDR cells frequently express other efflux proteins, such as MRP (Zaman et al, 1995; Hayes and Pulford 1995), have high levels of $H^+$-ATPase (Benderra et al, 1998) and exhibit the activated glutathione/glutathione S-transferase detoxification system (Altan et al, 1998). These and possibly other factors could impose high energy requirements in MDR cells and collectively contribute to the elevated responsiveness of these cells to Pluronic. The high responsiveness to P85 of the cell lines, which have relatively low levels of Pgp expression, HUVECs and Caco-2 may be one indication of the possible involvement of alternative mechanisms in the energy depletion.

In any case, it appears that energy depletion is an Achilles heel of MDR cells, turning the high energy requirements imposed on these cells by drug resistance mechanisms into a vulnerability resulting in elevated cytotoxic effects. While a number of experimental and clinical approaches have been studied to overcome MDR, including the use of MDR chemosensitizers, the appearance of several distinct transporters in resistant cells may limit the success of those agents, which target a single drug efflux pump. Furthermore, the combination of several independent mechanisms of drug resistance might complicate chemotherapy and reinforces the need for development of novel drugs and drug, formulations effective against drug-resistant cancers. It has long been suggested that a broadly successful strategy for killing drug-resistant cancer cells could be based on selective energy depletion in these cells, since many mechanisms of drug resistance are energy dependent (Mansouri et al, 1992). However, no study so far has reported such a significant decrease in ATP levels, which is observed selectively in MDR-expressing cells. Therefore, the finding of energy-depleting effects of Pluronic block copolymers, in combination with their very high sensitization effects and ability to inhibit multiple mechanisms of drug resistance in MDR cells is of considerable theoretical and practical significance.

### ACKNOWLEDGEMENT

This work was supported by the United States National Institutes of Health (RO1 NS366229; RO1 CA8922501).

### REFERENCES

Alakhov VYu, Moskaleva EYu, Batrakova EV and Kabanov AV (1996) Hypersensitization of multidrug resistant human ovarian carcinoma cells by pluronic P85 block copolymer. *Bioconjug Chem* **7**: 209–216

Alakhov V, Klinsky E, Li S, Pietrzynski G, Venne A, Batrakova E, Bronich T and Kabanov A (1999) Block copolymer-based formulation of doxorubicin. From cell screen to clinical trials. *Colloids and Surfaces B: Biointerfaces* **16**: 113–134

Altan N, Chen Y, Schindler M and Simon SM (1998) Defective acidification in human breast tumor cells and implications for chemotherapy. *J Exp Med* **187**: 1583–1598

Batrakova EV, Dorodnych TY, Klinskii EY, Kliushnenkova EN, Shemchukova OB, Goncharova ON, Arjakov SA, Alakhov VY and Kabanov AV (1996) Anthracycline antibiotics non-covalently incorporated into the block copolymer micelles: *in vivo* evaluation of anti-cancer activity. *Br J Cancer* **74**: 1545–1552

Batrakova EV, Han HY, Miller DW and Kabanov AV (1998) Effects of pluronic P85 unimers and micelles on drug permeability in polarized BBMEC and Caco-2 cells: *Pharm Res* **15**: 1525–1532

Batrakova E, Lee S, Li S, Venne A, Alakhov V and Kabanov A (1999) Fundamental relationships between the composition of Pluronic block copolymers and their hypersensitization effect in MDR cancer cells. *Pharm Res* **16**: 1375–1381

Batrakova EV, Miller DW, Li S, Alakhov VY, Kabanov AV and Elmquist WF (2001) Pluronic P85 enhances the delivery of digoxin to the brain: *in vitro* and *in vivo* studies. *J Pharmacol Exp Ther* **296**: 551

Benderra Z, Morjani H, Trussardi A and Manfait M (1998) Role of the vacuolar $H^+$-ATPase in daunorubicin distribution in etoposide-resistant MCF7 cells overexpressing the multidrug-resistance associated protein. *Int J Oncol* **12**: 711–715

Druekes P, Schinzel R and Palm D (1995) Photometric microtiter assay of inorganic phosphate in the presence of acid-labile organic phosphates. *Analytical Biochem* **230**: 173–177

Evers R, Zaman GJ, van Deemter L, Jansen H, Calafat J, Oomen LC, Oude Elferink RP, Borst P and Schinkel AH (1996) Basolateral localization and export activity of the human multidrug resistance-associated protein in polarized pig kidney cells. *J Clin Invest* **97**: 1211–1218

Evers R, Kool M, van Deemter L, Janssen H, Calafat J, Oomen LC, Paulusma CC, Oude Elferink RP, Baas F, Schinkel AH and Borst P (1998) Drug export activity of the human canalicular multispecific organic anion transporter in polarized kidney MDCK cells expressing cMOAT (MRP2) cDNA. *J Clin Invest* **101**: 1310–1319

Evers R, Kool M, Smith AJ, van Deemter L, de Haas M and Borst P (2000) Inhibitory effect of the reversal agents V-104, GF120918 and Pluronic L61 on MDR1 Pgp-, MRP1- and MRP2-mediated transport. *Br J Cancer* **83**: 366–374

Ferrari M, Fornasiero MC and Isetta AM (1990) MTT colorimetric assay for testing macrophage cytotoxic activity *in vitro*. *J Immunol Methods* **131**: 165–172

Fontaine M, Elmquist WF and Miller DW (1996) Use of rhodamine 123 to examine the functional activity of P-glycoprotein in primary cultured brain microvessel endothelial cell monolayers. *Life Sci* **59**: 1521–1531

Garewal HS, Ahmann FR, Schifman RB and Celniker A (1986) ATP assay: ability to distinguish cytostatic from cytocidal anticancer drug effects. *J Natl Cancer Inst* **77**: 1039–1045

Goldstein LJ, Gottesman MM and Pastan I (1992) Multidrug resistance in human cancers. *Crit Rev Oncol Hematol* **12**: 243–253

Hayes JD and Pulford DJ (1995) The glutathione S-transferase supergene family: regulation of GST and the contribution of the isoenzymes to cancer chemoprotection and drug resistance. *Crit Rev Biochem Mol Biol* **30**: 445–600

Iwahana M, Utoguchi N, Mayumi T, Goryo M and Okada K (1998) Drug resistance and P-glycoprotein expression in endothelial cells of newly formed capillaries induced by tumors. *Anticancer Res* **18**: 2977–2980

Kaplan O, Navon G, Lyon RC, Faustino PJ, Straka EJ and Cohen JS (1990) Effects of 2-deoxyglycose on drug-sensitive and drug-resistant human breast cancer cells: Toxicity and magnetic resonance spectroscopy studies of metabolism. *Cancer Res* **50**: 544–551

Kaplan O, Jaroszewski JW, Clarke R, Fairchild CR, Schoenlein P, Goldenberg S, Gottesman MM and Cohen JS (1991) The multidrug resistance phenotype: 31P nuclear magnetic resonance characterization and 2-deoxyglucose toxicity. *Cancer Res* **51**: 1638–1644

Kirillova GP, Mokhova EN, Dedukhova VI, Tarakanova AN, Ivanova VP, Efremova NV and Topchieva IN (1993) The influence of pluronics and their conjugates with proteins on the rate of oxygen consumption by liver mitochondria and thymus lymphocytes. *Biotechnol Appl Biochem* **18**: 329–339

Lee JS, Paull K, Alvarez M, Hose C, Monks A, Grever M, Fojo AT and Bates SE (1994) Rhodamine efflux patterns predict P-glycoprotein substrates in the National Cancer Institute drug screen. *Mol Pharmacol* **46**: 627–638

Mansouri A, Henle KJ and Nagle WA (1992) Multidrug resistance: prospects for clinical management. *SAAS Bull Biochem Biotechnol* **5**: 48–52

Miller DW, Fontaine M, Kolar C and Lawson T (1996) The expression of multidrug resistance-associated protein (MRP) in pancreatic adenocarcinoma cell lines. *Cancer Lett* **107**: 301–306

Rebbeor JF and Senior AE (1998) Effects of cardiovascular drugs on ATPase activity of P-glycoprotein in plasma mambranes and in purified reconstituted form. *Biochim Biophys Acta* **1369**: 85–93

Shepard RL, Winter MA, Hsaio SC, Pearce HL, Beck WT and Dantzig AH (1998) Effect of Modulators on the ATPase activity and vanadate nucleotide trapping of human P-glycoprotein. *Biochem Pharmacol* **56**: 719–727

Slepnev VI, Kuznetsova LE, Gubin AN, Batrakova EV, Alakhov VYu and Kabanov AV (1992) Micelles of poly(oxyethylene)-poly(oxypropylene) block copolymer

 *© 2001 Cancer Research Campaign*

(pluronic) as a tool for low-molecular compound delivery into a cell. Phosphorylation of intracellular proteins with micelle incorporated [$\gamma_t^{32}$P]ATP. *Biochem Internat* **26**: 587–595

Venne A, Li S, Mandeville R, Kabanov AV and Alakhov VYu (1996) Hypersensitizing effect of Pluronic L61 on cytotoxic activity, transport, and subcellular distribution of doxorubicin in multiple drug-resistant cells. *Cancer Res* **56**: 3626–3629

Zaman GJ, Lankelma J, van Tellingen O, Beijnen J, Dekker H, Paulusma C, Oude Elferink RP, Baas F and Borst P (1995) Role of glutathione in the export of compounds from cells by the multidrug-resistance-associated protein. *Proc Natl Acad Sci USA* **92**: 7690–7694

*© 2001 Cancer Research Campaign* 
